# Perceived privacy in home office and musculoskeletal complaints: a test of family–work conflict, work–family conflict, and relaxation as mediators

**DOI:** 10.1007/s43545-022-00553-y

**Published:** 2022-10-29

**Authors:** Milena Sina Wütschert, Diana Pereira, Andrea Eggli, Hartmut Schulze, Achim Elfering

**Affiliations:** 1 Institute of Psychology, University of Bern, Bern, Switzerland; 2 Institute of Social Work, University of Applied Science of Bern, Bern, Switzerland; 3 Institute for Research and Development of Collaborative Processes, FHNW University of Applied Sciences and Arts Northwestern of Switzerland, Olten, Switzerland

**Keywords:** Home office, Privacy, Musculoskeletal complaints, Family–work conflict, Work–family conflict, Relaxation

## Abstract

Many employees have had to telework all year during the COVID-19 pandemic. Even though working from home has many advantages, there are also some disadvantages worth to consider. Lack of privacy is a relevant factor when it comes to the development of severe musculoskelatal issues. This study investigated the link between perceived privacy in home office and musculoskeletal complaints (MSCs). Family–work conflict (FWC), work–family conflict (WFC), and relaxation were tested as potential mediators for the relationship between perceived privacy and MSCs. The present study’s questionnaire was filled out by 287 teleworking employees. Hypotheses were tested via multiple mediation analyses examining levels of perceived privacy in home office, and its relationship on MSCs. Furthermore, the underlying effect of FWC, WFC, and MSCs were tested with a structural equation model. As assumed, lack of privacy while working at home was linked to individuals more frequently experiencing MSCs. However, the structural equation model showed no significant mediation effect. Work design efforts must address privacy while employees perform telework at home to prevent MSCs.

## Introduction

The ongoing COVID-19 pandemic has forced employees around the world to telework part and full time from home. A variety of definitions exist for the term “telework” (Berg et al. 2021; Felstead and Jewson 2002; Jaakson and Kallaste 2010). Before the COVID-19 pandemic, telework was perceived as one of several types of work differing from traditional office work. Telework is characterized by employees’ increased use of information and communications technologies (ICTs), which allow users to work from anywhere and at any time (Berg et al. 2021; Messenger and Gschwind 2016). Many companies allowed employees to telework from home for one or two days per week. In the literature, consistent evidence has shown the positive effects teleworking on job performance, satisfaction (e.g., Bentley et al. 2013; de Menezes and Kelliher 2011; Kroll and Nuesch 2019), and absenteeism (e.g., Benach et al. 2012; Higgins et al. 2014; Joyce et al. 2010; Kossek et al. 2006).

Due to the current COVID-19 pandemic, many employees are now permanently working from home. Depending on their home environment, employees may not experience workplace flexibility or privacy because they must use what their home provides in terms of ergonomics and infrastructure, leading to a lack of perceived privacy. Therefore, the present study examined how levels of perceived privacy in home office affect influences the likelihood of employees’ experiencing musculoskeletal complaints (MSCs) while working from home and whether boundary management mediates the relationship between perceived privacy and musculoskeletal complaints (MSCs).

### The level of perceived privacy in home office as an environmental factor and MSCs

Stress research has mainly focused on the psychosocial factors that influence job performance, job satisfaction, strain, and employee health. However, some theoretical models of stress at work have included the physical environment as an additional factor (Haapakangas et al. 2014; Vischer and Wifi 2017). The person–environment fit (P–E fit) approach (Dewe et al. 2012) states that employees exist in a person–environment interaction system in which they continuously change their environment while adapting and adjusting their behavior to it (Dewe et al. 2012; Vischer and Wifi 2017). The Person–Environment Fit approach is comprehensive in that it can be applied in any context where the individual responds to the environment and the object within it. The model considers (1) the Need–Supplies Fit, that is, to what extend the design of the environment matches the personal needs and (2) the Demand–Ability Fit, that is, whether the demand of the environment matches the capabilities of the individual (Dewe et al. 2012; Vischer and Wifi 2017). Regarding to the home office, there is a knowledge gap concerning both fits. Currently, little is known in the literature about how the work environment at home is designed or individual living conditions look like (Hax-Noske 2019). According to Altman (1975), it is precisely the territoriality in the home office that can be challenging. The primary territory is in the permanent possession of one person (e.g., own workroom). It offers a high degree of intimacy and privacy, and the owner has complete power of disposition and access control. The secondary territories are used by several people. There is a shared control of access and disposition. The right of use may not be sufficiently clarified among the persons, which can lead to conflict potential. It can, therefore, be seen that it is precisely the second territory to which the home office can be assigned those harbors certain potential for conflict regarding the perceived privacy in home office. Vieira and Meirinhos (2021) and Wütschert et al. (2021) studies provided additional evidence that perceived privacy among home-based teleworkers may play a supporting role in mental health. The construct of perceived privacy is associated with disturbance and distraction and represents the perceived control of outside stimuli regarding to visual privacy and acoustic privacy (Wohlers and Hertel 2017; Elsbach and Pratt 2007; Haapakangas et al. 2018a, b). The literature shows that perceived lack of privacy is a significant stress factor (Pejtersen et al. 2006; Danielsson and Bodin 2009; Haapakangas et al. 2018a, b). Perceived lack of privacy can facilitate acute stress reactions and can be related to mental and physical health issues (Haapakangas et al. 2018a, b; Lee and Brand 2010; Haapakangas 2014). Work strain includes the psychological (emotional and cognitive), behavioral (fight and flight), and physiological (autonomic and neuroendocrine functions) reactions to work demands (work stressors) (Allen et al. 2017; Ganster and Rosen 2013). Work strain, in turn, is also related to MSCs. MSCs include injuries and disorders of the muscles, nerves, tendons, ligaments, joints, cartilage, and spinal disks (Burton and WHO 2010). Repetitive strain injuries, also known as cumulative trauma disorders, are also a type of MSCs. These disorders are not immediately apparent and can take days, months, or years before they affect an employee (Burton and WHO 2010). Eatough et al. (2012) showed that work strain mediated the relationship between work stressors and work-related MSCs (Lundberg and Melin 2002). Several studies have shown that work strain causes muscle tension triggered by mental (Elfering 2006; Elfering and Mannion 2008; McFarlane 2007) or physical stress (Elfering et al. 2002, 2008). While Aegerter et al. (2021) study found no evidence of an increase in employee neck pain during the COVID-19 pandemic, their results highlighted the effect psychosocial factors have on teleworkers. Aegerter et al. (2021) suggested that further studies are needed to clarify these psychosocial factors’ influence.

### The underlying mechanisms among the relationship between the level of perceived privacy in home office and MSCs

### Boundary management and recovery

As Eatough et al. (2012) postulated, job-related stressors, such as perceived privacy in Ome Offices, may have more complex effects on MSCs beyond simple bivariate relationships, suggesting that there are underlying mechanisms involved. There are already multiple theoretical models speculating the mechanisms underlying the association between job-related stressors and MSC; however, the results remain inconsistent (Eatough et al. 2012). Thus, more research investigating the underlying mechanisms is needed. In this paper, we suppose that boundary management and relaxation are crucial in the relationship between perceived privacy in home office and MSCs.

Due to the COVID-19 pandemic, boundaries have disappeared in employee’s homes, leading work, and family activities to occur in the same space on a permanent basis. This situation makes boundary management more challenging (Allen et al. 2021). Boundary management is defined as the way individuals create, maintain, and change boundaries to navigate the world, including their work and private roles (Allen et al. 2021; Ashforth et al. 2000; Kreiner et al. 2009). Boundary theory is rooted in organization role theory (Biddle 1986; Kahn et al. 1964; Katz and Kahn 1978). Katz and Kahn (1978) define an organization as an open system of roles. Boundaries that are related to work and family can be delineated by the expected behaviors for each role, which determines how individuals manage these boundaries. However, when it is difficult to transition between roles, boundaries can be a source of conflict. Inter-role conflict occurs when role pressures associated with membership in one group conflict with role pressures related to membership in another group. Greenhaus and Beutell (1985) define family–work conflict (FWC) and work–family conflict (FWC) as “a form of inter-role conflict in which work, and family roles are not aligned in some respect” (p. 77).

Spending extended time in a home office leads boundaries to disappear between work and private life. The disappearance of these boundaries impacts not only work but also recovery. Demerouti et al. (2007) define recovery as “the sense of urgency that people feel to take a break from their demands when fatigue is building up” (p. 2). Typical indicators of the need for recovery are employees’ finding it difficult to relax at the end of a working day, requiring days off to rest and feeling tired when they start a new working day. Sonnentag and Fritz (2007) characterize relaxation as characterize relaxation as a period of low activation with positive effects on mental and physical well-being. Meijman et al. (1992) effort–recovery theory attempts to explain the importance of relaxation. Its main premise is that effort expenditure at work is associated with stress responses. In optimal conditions, stress responses return to pre-stressor levels during off-hours, and employees completely recover before the start of the next day. However, if stress reactions persist or recur during leisure time, the recovery phase cannot be completed, which not only influences people’s health but also their everyday behavior. For example, when people are not fully recovered, they find it difficult to fulfill their general responsibilities at work and in social life (Demerouti et al. 2007). The disappearance of boundaries between work and home suggests that perceived privacy may be associated with relaxation.

Demerouti et al. (2007) emphasized in their longitudinal study that stress experienced in the home environment spill over into and influence participation in one’s work environment. Conflicts are characterized by a spillover of negative emotions from one area into another. Home-based teleworkers’ lack of privacy may affect work performance and recovery, which, from a long-term perspective, may increase the likelihood of developing MSCs (Sonnentag 2018). The question about the role of FWC, WFC, and relaxation in the relationship between perceived privacy in home office and MSCs also arose. We hypothesized that FWC, WFC, and relaxation act as mediators for this relationship.

## Methods

### Purpose

**Fig. 1:**
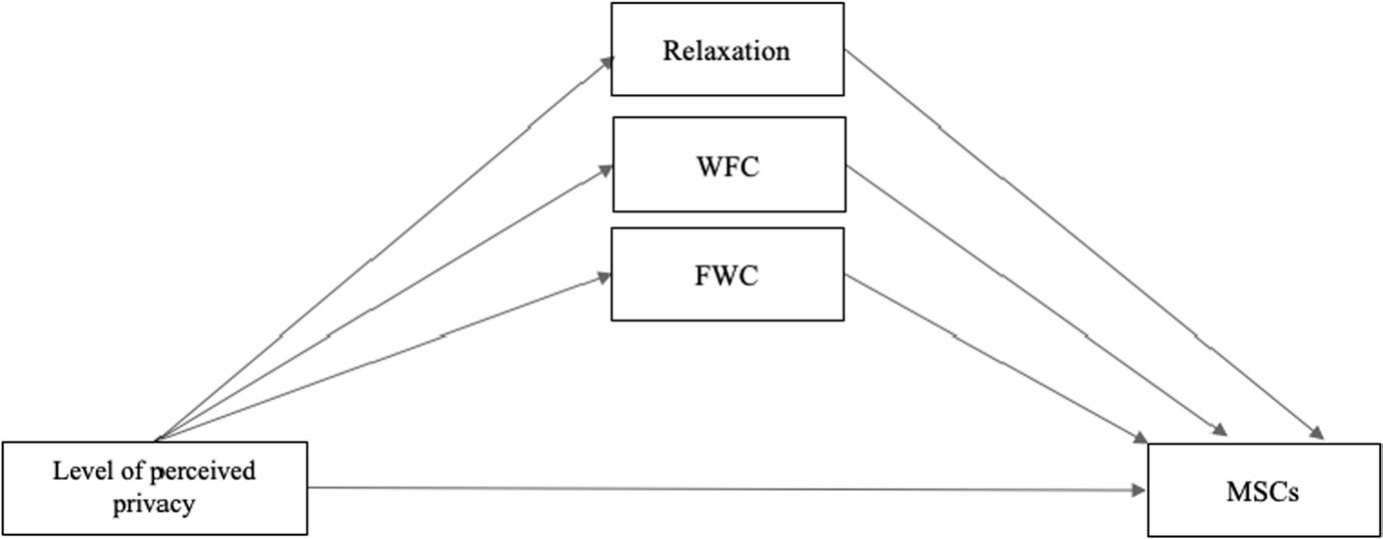
Mediation model: level of perceived privacy during home-based telework

In light of the ongoing COVID-19 crisis and resulting developments, the relationship between boundary management regarding work’s interference with family, family’s interference with work, and associated health effects should be explored in more detail. According to our knowledge, there are no published studies about how perceived privacy in home office is related to MSCs and about the underlying mechanisms. Furthermore, Demerouti et al. (2007) emphasized in their longitudinal study that stress experienced in the home environment spill over into and influence participation in one’s work environment. Conflicts are characterized by a spillover of negative emotions from one area into another. Home-based teleworkers’ lack of privacy may affect work performance and recovery, which, from a long-term perspective, may increase the likelihood of developing MSCs (Pereira and Elfering 2014; Sonnentag 2018). The question about the role of FWC, WFC, and relaxation in the relationship between perceived privacy in home office and MSCs also arose. We, therefore, hypothesized that perceived privacy is related to MSCs and that there are substantial underlying mechanisms (WFC, FWC, relaxation) that mediate the relationship between perceived privacy in home office and MSCs. The present cross-sectional study is intended to explore these relationships. The following hypotheses were formulated for the population of home-based teleworkers. Figure 1 visualizes the hypotheses in a mediation model.

H1: The level of perceived privacy is related to MSCs.
H2: Underlying mechanisms mediate the relationship between perceived privacy and MSCs.
H2a: FWC mediates the relationship between perceived privacy and MSCs.
H2b: WFC mediates the relationship between perceived privacy and MSCs.
H2c: Relaxation mediates the relationship between perceived privacy and MSCs.

### Procedure

The present study used a cross-sectional online survey that was distributed in Switzerland. The survey language was German. Participants were recruited through advertisements in magazines and on websites, as well as through social media such as LinkedIn (convenience sampling). This approach allowed us to recruit home-based teleworkers from different sectors. Answering the questionnaire online was time efficient for the participant. Prior to participation, all subjects were informed about the content of the study. They were told that participation is voluntary and that they could cancel at any time. They were further informed that the data would be used for scientific purposes only and would be stored and analyzed anonymously. We used Qualtrics as the platform to host the online survey. The data were collected over a three-month period from January to March 2021. This time span fell into the COVID-19 lockdown when Switzerland’s government recommended that people work from home. Due to convenience sampling, the participation rate could not be derived. Ethical approval (12.01.21, Ethics No. 2021-01-00001) was obtained from the Ethics Commission of the University of Bern, Switzerland prior to data collection. Only subjects between the age of 18 and 65 that telework a minimum of 1–2 days per week from home and possess age-appropriate health were included in the study.

### Sample

The age of the participants ranged between 18 to 65 years (M 3.19, SD 0.99). The most common age category was 40–59 years (35.8%). Of the total number of participants, 193 (65%) were female, and 103 (35%) were male. Regarding relationship status, 115 (39%) of the respondents were married, 110 (37%) reported they were in a committed relationship, 50 (17%) were single, and 18 (7%) were separated or divorced. Among all participants, 145 (49%) had full-time employment, 231 participants (78%) attended higher education, such as university, and 65 (22%) had participated in an apprenticeship.

Regarding the family situation, 172 (58%) had no children, and 124 (42%) had children. Regarding their home office situation, 184 (63.5%) participants had a separate office room in their home while 112 (36.5%) did not. Silent work was done by 182 respondents (68%), 127 (43%) participants worked on a laptop, while 98 (33%) had a monitor, keyboard, and mouse in their home office. Fifty (17%) participants stated that they appreciated their home office because they could balance family and work, and 65 (22%) appreciated having time flexibility. As to the reasons for a positive attitude towards home office, 44 (15%) of the respondents preferred working in a home office mainly because they experienced increased concentration, 71 (24%) preferred their home office to avoid conflicts at work, and 65 (22%) of respondents preferred to be alone when working.

### Measures

#### The perceived level of privacy in the home office

Distractions and disturbances caused by lack of privacy in one’s home office were measured with four items. Originally, these items have been introduced for usage in activity-based work settings, but we adapted them for the home office setting. An example item was “How satisfied are you with the visual distinction of your home office, for example, the visibility by others, seeing others, being seen?” The items were answered on a five-point Likert scale ranging from 1 (not satisfied at all) to 5 (totally satisfied). Cronbach’s alpha in the present data was 0.88. Prior studies have already used the privacy sub-scale to validate self-rated productivity and well-being ( et al. 2018a, b).

#### Relaxation experience

Relaxation experience was measured with one single item “In general, I can sit back and relax in my free time” from Sonnentag and Fritz’ (2007) Recovery Experience Questionnaire. This item was answered on a five-point Likert scale ranging from 1 (strongly disagree) to 5 (strongly agree). In the scale manual from Sonnentag and Fritz (2007), this item had the highest loading on the factor (p.213); therefore, it can be assumed that it is a valid item.

#### Work–family conflict and family–work conflict

WFC and FWC were measured with Netemeyer et al. (1996) scale, which has three items each. One item that evaluated WFC was “The demands of my work interfere with my home and family life.” The WFC items were answered on a five-point Likert scale ranging from 1 (strongly disagree) to 5 (strongly agree). Cronbach’s alpha in the present data was 0.80. One item that evaluated FWC was “The demands of my home and my family life interfere with work-related activities.” The FWC items were also answered on a five-point Likert scale ranging from 1 (strongly disagree) to 5 (strongly agree). Cronbach’s alpha in the present study was 0.85. This scale has been applied and validated in a variety of work–life balance studies (Labrague et al. 2021; Rupert et al. 2012).

#### Musculoskeletal complaints

MSCs were measured with four items from the German version of the Cornell Musculoskeletal Discomfort Questionnaire (D-CMDQ) (Kreuzfeld et al. 2016). An example item was “During the last work week, how often did you experience aches, pains, or discomfort in (1) neck, (2) shoulders, (3) upper back, and (4) lower back.” These items were answered on a five-point Likert scale ranging from 1 (never) to 5 (several times every day). Cronbach’s alpha in the present study was 0.80. Prior studies have used the D-CMDQ, and it has shown validity regarding the measurement of workplace ergonomic conditions among computer workers (Osama et al. 2018; Vahdatpour et al. 2019).

#### Control variables

The present study controlled for age (1 ≤  20 years; 2 = 20–29 years; 3 = 30–39 years; 4 = 40–59 years, 5 = 60–65 years) and gender (0 = male; 1 = female) because past studies have shown that age and gender affect MSCs in employees (Baur et al. 2018; Elfering et al. 2016). To take individual requirements into account, this study also controlled for full- or part-time employment, as suggested by Elfering et al. (2016).

### Data analysis

We performed data analysis using R software 4.0.2.. The strength of linear relationships between continuous variables was measured with a Pearson’s product-moment correlation. For the analysis, we used the packages lavaan and MVN, and for visualization, we used the packages semPlot and sjPlot.

To test our hypotheses, we conducted multiple mediation analyses with level of perceived privacy as the predictor FWC as well as WFC as mediators and MSCs as the dependent. Gender, age, and full- or part-time job were also entered into each model as control variables. The multiple mediation analysis was conducted following Preacher and Hayes (2004) recommendations, which included the steps by Baron and Kenny (1986) and estimated direct and indirect effects via bootstrapping, which does not require the assumption that the error is normally distributed (Preacher and Hayes 2004). When using the bootstrapped CI (lower limit of the CI [LL]; upper limit of the CI [UL]) procedure, mediation is indicated by the exclusion of zero from the CI for the indirect effect. If the bootstrapped CI does not include zero, then the mediating effect differs significantly from zero (Preacher and Hayes 2004). In this study, we estimated a 95% bias-corrected CI using 5,000 bootstrapped samples.

### Additional analysis

Self-reported measures and cross-sectional studies are susceptible to common method bias (CMB). Podsakoff et al. (2012) recommended Harman’s one-factor test, in which all items measuring latent variables are loaded into one common factor. If the total variance for a single factor is less than 50%, it is concluded that CMB does not affect the results. The first factor in the present study explained a total variance of 20%, so no common method bias was observed.

## Results

### Descriptive statistics and correlations

Means and standard deviations for all relevant variables and bivariate correlations are reported in Table 1. The level of perceived privacy in home office was negatively related to MSCs [*r*(287) = − 0.252, *p* < 0.01] and FWC (*r* = − 0.170, *p* < 0.01). Relaxation was negatively associated with MSCs (*r* = − 0.204, *p* < 0.01), FWC (*r* = − 0.244, *p* < 0.01) and WFC (*r* = − 0.399, *p* < 0.01). Age was positively related to the level of perceived privacy in the home office (*r* = 0.246, *p* < 0.01). Men had significantly more MSCs (*r* = − 0.182, *p* < 0.01) than women and a significantly lower level of perceived privacy (*r* = 0.142, *p* < 0.05). Part-time work was positively associated with the level of perceive privacy in home office (*r* = 0.163, *p* < 0.01).

**Table 1:** Descriptive statistics and Pearson correlations

	Mean	SD	1	2	3	4	5	6	7	8
1. Privacy in HO	3.927	1.032	1							
2. MSCs	1.805	0.758	− 0.252**	1						
3. FWC	1.342	0.491	− 0.170**	0.007	1					
4. WFC	1.860	0.788	− 0.048	0.066	0.319**	1				
5. Relaxation	3.736	0.966	0.094	− 0.204**	− 0.244**	− 0.399**	1			
6. Part-time work	8.494	1.857	0.163**	− 0.013	− 0.046	0.077	− 0.045	1		
7. Age	3.199	0.993	0.246**	− 0.045	0.041	0.193**	− 0.033	− 0.025	1	
8. Sex ^a^	0.347	0.477	0.142*	− 0.182**	0.084	− 0.033	0.023	0.291**	0.168**	1

*N* = 287

*HO* home office; *MSCs* musculoskeletal complaints; *FWC* family–work conflict; *WFC* work–family conflict

*p < 0.05, **p < 0.01, ***p < 0.001, two tailed

^a^0 = male, 1 = female

### Confirmatory factor analysis

The data were not normally distributed; therefore, we used the bias-corrected 5,000 bootstrapped method (Preacher and Hayes 2004). Due to the non-normal distribution, the robust maximum likelihood estimation (MLE) was used to calculate a confirmatory factor analysis (CFA). The CFA was conducted to examine convergent and discriminant validity. The convergent validity is the degree of confidence that a trait is measured well by its indicators (Campbell and Fiske 1959). Discriminant validity evaluates the extent to which each construct in the model differs from the other constructs (Bagozzi et al. 1991). The convergent validity of the measurement model can be evaluated by the average variance extracted (AVE) and the composite reliability (CR). AVE measures the level of variance captured by a construct versus the level caused by measurement error. Values above 0.7 are considered very good, whereas 0.5 is acceptable (Fornell and Larcker 1981). CR is a less biased estimate of reliability than Cronbach’s alpha; the acceptable values of CR are 0.7 and above (Bagozzi et al. 1991). All constructs exceeded the recommended values. Table 2 shows evidence for the convergent and discriminant validity of all the reflective latent constructs.

**Table 2:** CFA model: convergent and discriminant validity

Construct	*R*^2^ Estimate	Standardized loading (*p*)	CR	AVE	$\alpha$
Privacy HO			0.87	0.63	0.88
Privacy1	0.63	0.80***			
Privacy2	0.53	0.73***			
Privacy3	0.65	0.80***			
Privacy4	0.72	0.85***			
FWC			0.80	0.57	0.85
FWC1	0.64	0.80***			
FWC2	0.53	0.73***			
FWC3	0.56	0.75***			
WFC					
WFC1	0.49	0.70***	.86	0.67	0.80
WFC2	0.84	0.92***			
WFC3	0.60	0.83***			
MSCs			0.82	0.53	0.80
MSCs1	0.67	0.82***			
MSCs2	0.64	0.80***			
MSCs3	0.58	0.76***			
MSCs4	0.24	0.50***			

*N* = 287

*CR* composite reliability; *AVE* average variance extracted; $\alpha$ Cronbach’s alpha; *HO* home office; *FWC*  family–work conflict; *WFC* work–family conflict; *MSCs* musculoskeletal complaints

*p < 0.05, **p < 0.01, ***p < 0.001, two-tailed

The CFA was also used to assess measurement model fit. To judge how well the model represented the data, fit indices such as the *χ*^2^ statistic, the non-normed fix index (NNFI), the comparative fit index (CFI), and the standardized root-mean-square residual (SRMR) were used. A normed *χ*^2^ should be lower than 3.0 (Malkanthie, 2018). For NNFI, values larger than 0.95 or 0.97 constitute a good fit with an NNFI near 1 represents a perfect fit. For CFI, values larger than 0.95 constitute a good fit, and values above 0.90 mark an acceptable fit (Medsker et al. 1994). For the SRMR, it has been suggested that values below 0.05 constitute a good fit, while values in the 0.05 to 0.10 range are an acceptable fit (Browne and Cudeck, 1992). The measurement model provided a good fit for the data [*χ*^2^ (71) = 1.26 (*n* = 269), *p* < 0.001; NNFI = 0.97; CFI = 0.98; SRMR = 0.04]. Further fit indices of the user model and baseline model are shown in Table 3. The CFA user model supported the hypothetical factor structure better than the CFA baseline model.

**Table 3:** Goodness of fit statistics

	*χ*^2^^a^	*df*	*p*	CFI^b^	RMSEA^c^	AIC^d^
CFA: user model	106.289	71	< 0.000	0.980	0.041	202.289
CFA: baseline model	1847.439	91	< 0.000	0.000	0.256	1903.439

*N* = 287

*χ*^2^ chi-square value; *df* degrees of freedom; *p* p value of minimum discrepancy; *CFI* comparative fit index; *RMSEA* root–mean-square error of approximation; *AIC* aikaike information criterion

^a^The model is considered as fit to the data if the *χ*^2^ value is low relative to the degree of freedom with an insignificant *p* value (*p* < 0.05) (Malkanthie^2018^)

^b^The comparative fit index (CFI) > 0.90 reflect an acceptable fit between the model and the data (Malkanthie 2018)

^c^Root-Mean-Square Error of Approximation (RMSEA) value < 0.05 reflects a good fit of the model (Schermelleh-Engel et al. 2003). RMSEA is a measure of fit that considers the population moments rather than sample moments

^d^Aikaike information criterion (AIC) should be as low as possible in model comparing

(Malkanthie 2018)

The standardized path coefficient $\beta$ from the CFA showed the relationship between the constructs. There was a significant relationship between the level of perceived privacy in the home office and FWC ($\beta$ = − 0.183, *p* = 0.010), level of perceived privacy in the home office, and MSCs ($\beta$ = − 0.268, *p* < 0.001) as well as WFC and FWC ($\beta$ = 0.354, *p* < 0.001). The level of perceived privacy in the home office and WFC ($\beta$ = − 0.026, *p* = 0.692), FWC, and MSCs ($\beta$ = − 0.015, *p* = 0.829) as well as WFC and MSCs ($\beta$ = 0.058, *p* = 0.390) were not significant.

### Test of direct and indirect paths

The test of direct paths showed that the level of perceived privacy in the home office was negatively related to MSCs and that this effect was significant ($\beta$ = − 0.18, SE = 0.04, *p* < 0.001). The relationship of perceived privacy in the home office to FWC was significantly negative ($\beta$ = − 0.11, SE = 0.03, *p* < 0.001). The relationship of FWC to MSCs was not negative, but not significant ($\beta$ = − 0.04, SE = 0.10, *p* = 0.282). The relationship between the level of perceived privacy in the home office and WFC was significantly negative ($\beta$ = − 0.09, SE = 0.05, *p* = 0.004), and the relationship between WFC and MSCs was positive, but not significant ($\beta$ = 0.03, SE = 0.06, *p* = 0.662). The level of perceived privacy in the home office had a positive but non-significant relationship with relaxation ($\beta$ = 0.11, SE = 0.06, *p* = 0.070). The relationship between relaxation and MSCs was significantly negative ($\beta$ = − 0.15, SE = 0.05, *p* < 0.002). Table 4 gives and overview over all direct effects.

**Table 4:** Regression results for multiple mediation

Direct and total effects	$\beta$	SE	*t*	*p*
Privacy HO → MSCs	− 0.18***	0.04	− 4.09	0.000
Privacy HO → Relaxation	0.11	0.06	1.81	0.070
Privacy HO → FWC	− 0.11***	0.03	− 3.63	0.001
Privacy HO → WFC	-0.09**	0.05	− 1.88	0.004
Relaxation → MSCs	− 0.15**	0.05	− 3.06	0.002
FWC → MSCs	− 0.04	0.10	− 1.08	0.282
WFC → MSCs	0.03	0.06	0.44	0.662

Bootstraps results for indirect effect	*M*	SE	LL 95% CI	UL 95% CI
Indirect Effect on Relaxation	− 0.02	0.01	− 0.04	0.00
Indirect Effect on FWC	0.00	0.01	− 0.02	0.03
Indirect Effect on WFC	− 0.00	0.01	− 0.02	0.01

*N* = 287, Bootstrap size = 5000

*LL*  lower limit; *CI*  confidence interval; *UL*  upper limit; *HO*  home office; *MSCs*  musculoskeletal complaints; *FWC*  family–work conflict; *WFC*  work–family conflict

*p < 0.05, **p < 0.01, ***p < 0.001, two-tailed

**Fig. 2:**
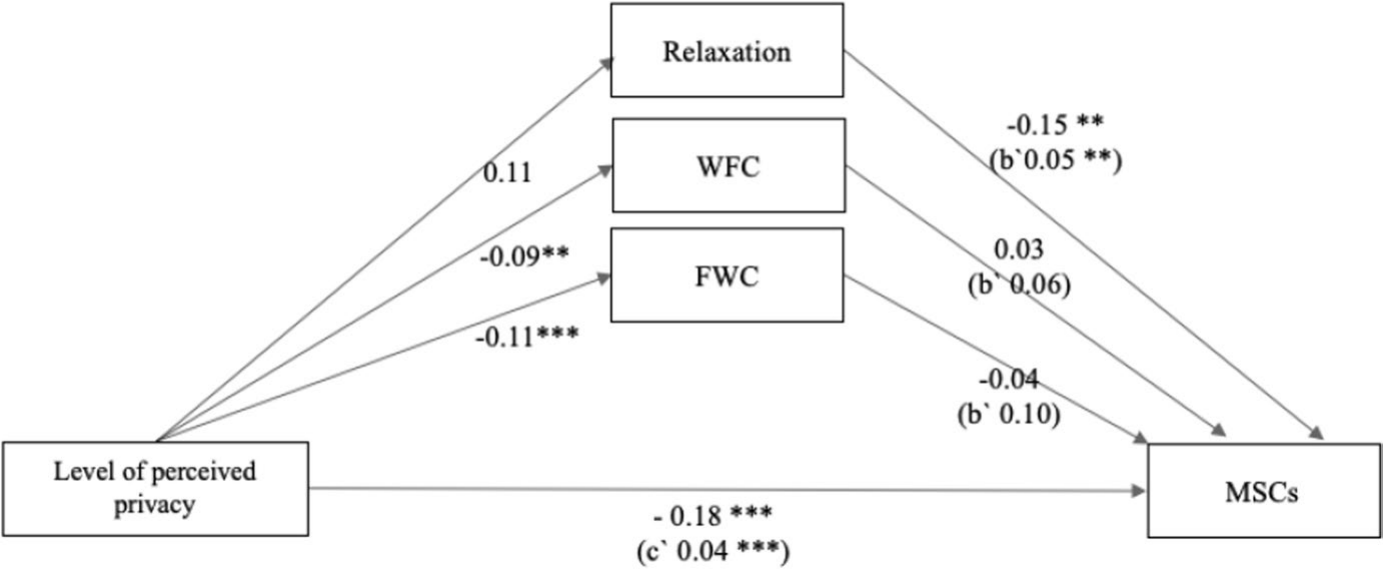
Mediation model: level of perceived privacy during home-based telework. Note. N = 287. Standardized Coefficients are reported. Included control variables: age, gender, and part-time work. * *p* < 0.05, ** *p* < 0.01, *** *p* < 0.001, two-tailed

To see whether there were significant indirect effects, we performed a multiple mediation analysis. FWC (*M* = 0.00, CI = [− 0.02, 0.03]), WFC (*M* = − 0.00, CI = [− 0.02, 0.01]), and relaxation (*M* = − 0.02, *CI* = [− 0.04, 0.00]) did not act as mediators, as shown in Table 4. Figure 2 provides an overview over the present hypotheses and findings.

## Discussion

The present study investigated the impact of the level of perceived privacy in home office on MSCs. Additionally, we examined the mediating effects of relaxation, FWC and WFC on the relationship of perceived privacy and MSCs. The data showed significant evidence that the level of perceived privacy in the home office has a negative association with MSCs. Consistent with our expectation, the evidence supports our assumption that the level of perceived privacy in the home office (e.g., opportunities to retreat) is a predictor of physical health. Thus, Hypothesis 1 was supported. This finding is in line with Vischer’s (2007) environmental comfort model, which postulates that a balanced fit between employee (the demand of the environment matches with the abilities and skills of the individual) and workplace environment (appropriate workplace design) puts employees’ workloads into perspective, and thus, counteracts workplace stress (Dewe et al. 2012; Vischer and Wifi 2017). The level of perceived privacy in the home office belongs to Vischer’s (2007) third category, psychological comfort. Psychological comfort is defined as a feeling of belonging, ownership, and control over one’s workspace. Psychological comfort entails the concept of subjectively experienced privacy (Vischer 2007). One possible explanation for why a high level of perceived privacy in the home office led to low MSCs is that perceived privacy can be influenced by the design of the physical workplace, such as physical features that separate the space between working and living environment (Fonner and Stache 2012; Kossek et al. 2006; Wohlers and Hertel 2017). When appropriating and controlling space, employees can change the meaning of a place in their home according to their interests (appropriation process). During the appropriation process, the employee’s behavior in the space is determined and defined by the employee (Wapshott and Mallett 2011). This control provides the individual with a sense of security, which in turn reduces perceived stress in the workplace (Vischer 2007; Wapshott and Mallett 2011). Perceived control over one’s work environment is assumed to reduce the impact of work stress and is positively associated with social relationships, environmental satisfaction, and job satisfaction (Vischer 2007). In the context of open workplace design, several studies highlighted the positive effects of high perceived privacy and well-being (Haapakangas et al. 2018a, b; Haapakangas et al.  2014; Hongisto et al. 2016).

Although FWC, WFC, and relaxation do not act as mediators, the present paper shows that relaxation acts as an independent predictor of MSCs whereas the level of perceived privacy acts as an independent predictor of FWC and WFC among homebased teleworkers. How can the observed lack of mediation be explained? A possible explanation for the non-significant mediation is provided by Wapshott and Mallett (2011). The authors emphasized that a person who can acquire a separate room or at least a certain area to work at home can find a symbolic mechanism to cognitively detach the connection between work and family, while other members of the family can recognize this room as a work zone. As a result, individuals experience fewer distractions, and others who share the environment understand the separation and follow specific rules to respect the individual’s space (Wapshott and Mallett 2011). Additionally, Solís (2016) showed in his research that increasing one’s number of teleworking days per week led to a reduction in family and work interference. This result is consistent with Hill et al. (2003) and Joyce et al.’s (2010) findings. Solís (2016) concluded that it is possible that the longer employees work from home, the better they can organize their time and develop strategies to avoid conflicts between work and family. According to Altman's (1975) theory, it can be assumed that after a certain period of time, a consensus with the other family members on the right of use of the shared (space) areas will arise. Present sample was consisted of employees who have been working in homoeffice for at least 1 year. Thus, they may have already adapted in relation to the stressors (e.g., conflicts related to work and family) of their specific working environment in home office.

## Limitations

Our results should be interpreted with caution for several reasons.

First, it is well known from the literature that MSCs may not become immediately apparent in those suffering from them. It can take days, months, or even years for MSCs to affect a worker (Burton and WHO 2010). A sample that shows more variance in terms of FWC, WFC, and relaxation may lead to different results.

Second, the present study used self-reported measures and a cross-sectional design. Cross-sectional studies are sensitive to CMB. We controlled for CMB with the Herman factor, yet this study’s results were not supported with objective or extended data, such as interviews.

Third, in our sample, 184 (62%) of participants had their own workplace, and 231 (78%) worked in colleges and universities. In Switzerland, most academic institutions and their employees are advanced and knowledgeable about flexible working conditions.

Fourth, the present study is limited to Switzerland. The context of telework in other countries is not covered here. It is difficult to compare other countries, especially in the case of teleworking (Eurofound 2020). The progress of telework depends on the respective working conditions, which are handled differently from country to country (Kotera and Correa Vione 2020). Technological progress also necessarily differs between countries and must be considered, especially regarding teleworking (Eurofound 2020; Hosoda 2021; Morrison et al. 2019).

## Implication

This study’s results underline the importance of environmental factors in establishing privacy in the home office. To avoid the consequences of workplace stress, workplace design must be adapted to the individual needs of each employee through ergonomic, organizational, and personal support by their employers (Mojtahedzadeh et al. 2021; Parker and Turner 2002; Parker et al. 2017). Employers should play an advisory role for employees to establish working conditions in their home offices and acquire self-management and work design skills, such as self-leadership and responsibility for the structuring of one’s work activities and motivational strategies (Dettmers and Clauß 2018; Mojtahedzadeh et al. 2021). Parker et al. (2017) emphasized that work design impacts individual work performance in four key categories of psychological mechanisms: motivation, knowledge, skill, and opportunity. These categories can support employees in learning to distance themselves from work, even when environmental factors are not optimal. Furthermore, breaks from work should not be spent on work-related activities. To enhance work detachment and avoid physical tension, employees should be as active as possible during breaks by exercising, stretching, or practicing progressive muscle relaxation or mindfulness exercises. To reduce possible role conflicts, workers should create time and space boundaries between their work and private life. If space is not available, then employees should consciously change their location during breaks. Work detachment can also be supported through fixed rituals before and after working (Mojtahedzadeh et al. 2021; Mustafa and Gold, 2013; Syrek et al. 2017). In today’s world, it cannot be assumed that every employee has his or her own room to work. Therefore, innovative approaches must be developed to show how privacy can be maintained in the home office with physical features and non-physical features, such as mental strategies. Sonnentag (2018) suggested that initiating processes that stimulate recovery (e.g., relaxation) is a powerful approach to counteracting the negative effects of job stressors. Therefore, these processes should be more thoroughly examined. Sonnentag (2018) emphasized the importance of prioritizing recovery, especially because people who experience high levels of job stressors tend to not detach from work during non-work time [e.g., engage in less physical activity and have poor sleep quality; see also the meta-analysis from Sonnentag (2018)]. Furthermore, little research has been conducted regarding and the question how resources such as social support from supervisors (Chen et al. 2021; De Bloom and Keller 2021) or individual coping strategies (Chang et al. 2021; Wang et al. 2020) influence working from home. But precisely because the influence of these topics is so important for the design of future work models, they should be investigated.

Future research must be conducted to examine how a high level of perceived privacy can be created in the home office. In today’s world, it cannot be assumed that every employee has his or her own room to work. Therefore, innovative approaches must be developed to show how privacy can be maintained in the home office with physical features and non-physical features, such as mental strategies. Sonnentag (2018) suggested that initiating processes that stimulate recovery (e.g., relaxation) is a powerful approach to counteracting the negative effects of job stressors. Therefore, these processes should be more thoroughly examined. Sonnentag (2018) emphasized the importance of prioritizing recovery, especially because people who experience high levels of job stressors tend to not detach from work during non-work time [e.g., engage in less physical activity and have poor sleep quality; see also the meta-analysis from Sonnentag (2018)]. Furthermore, little research has been conducted regarding and the question how resources such as social support from supervisors (Chen et al. 2021; De Bloom and Keller, 2021) or individual coping strategies (Chang et al. 2021; Wang et al. 2020) influences working from home. But precisely because the influence of these topics is so important for the design of future work models, they should be investigated in the future.

## Conclusion

The present study investigated the link between perceived privacy in home offices and MSCs. Furthermore, FWC, WFC, and relaxation were tested as potential mediators for the relationship between perceived privacy and MSCs. The significant results show that a lack of privacy while working at home was linked to individuals more frequently experiencing MSCs. This study is making an important contribution to the field of work and organizational psychology. To the best of our knowledge, the impact of the level of perceived privacy on home-based teleworkers has not yet been investigated in relation to MSCs.

## Data Availability

The datasets are available from the corresponding author on reasonable request.
